# Bicuspid Aortic Valve Disease and Ascending Aortic Aneurysms: Gaps in Knowledge

**DOI:** 10.1155/2012/145202

**Published:** 2012-10-31

**Authors:** Katie L. Losenno, Robert L. Goodman, Michael W. A. Chu

**Affiliations:** ^1^Department of Anatomy & Cell Biology, University of Western Ontario, London, ON, Canada N6A 5C1; ^2^Division of Cardiac Surgery, Department of Surgery, Lawson Health Research Institute, University of Western Ontario, London, ON, Canada N6A 5A5; ^3^B6-106 University Hospital, London Health Sciences Centre, 339 Windermere Road, P.O. Box 5339, London, ON, Canada N6A 5A5

## Abstract

The bicuspid aortic valve is the most common congenital cardiac anomaly in developed nations. The abnormal bicuspid morphology of the aortic valve results in valvular dysfunction and subsequent hemodynamic derangements. However, the clinical presentation of bicuspid aortic valve disease remains quite heterogeneous with patients presenting from infancy to late adulthood with variable degrees of valvular stenosis and insufficiency and associated abnormalities including aortic coarctation, hypoplastic left heart structures, and ascending aortic dilatation. Emerging evidence suggests that the heterogeneous presentation of bicuspid aortic valve phenotypes may be a more complex matter related to congenital, genetic, and/or connective tissue abnormalities. Optimal management of patients with BAV disease and associated ascending aortic aneurysms often requires a thoughtful approach, carefully assessing various risk factors of the aortic valve and the aorta and discerning individual indications for ongoing surveillance, medical management, and operative intervention. We review current concepts of anatomic classification, pathophysiology, natural history, and clinical management of bicuspid aortic valve disease with associated ascending aortic aneurysms.

## 1. Introduction

 The bicuspid aortic valve (BAV) is the most common congenital cardiac anomaly in developed nations. It has been presumed that the bicuspid morphology of BAV disease is largely responsible for valvular dysfunction and subsequent hemodynamic derangements. However, the clinical presentation of BAV disease remains quite heterogeneous with patients presenting from infancy to late adulthood with predominantly aortic stenosis, aortic insufficiency, or mixed lesions and variable associated abnormalities including hypoplastic left heart structures, aortic coarctation, and ascending aortic aneurysms. Emerging evidence suggests that the heterogeneous presentation of BAV phenotypes may be a more complex matter related to congenital, genetic, and/or connective tissue abnormalities. Currently, the etiology of aortic dilatation in patients with BAV disease remains unclear and as a result, management of these aortic aneurysms remains controversial. 

 Optimal management of patients with BAV disease and associated ascending aortic aneurysms often requires a thoughtful approach, carefully assessing various risk factors of the aortic valve and the aorta and discerning individual indications for ongoing surveillance, medical management, and operative intervention. Current guidelines recommend prophylactic replacement of the ascending aorta in patients with specific risk factors; however, the extent of the aortic resection remains debated. We review current concepts of anatomic classification, pathophysiology, natural history, and clinical management of BAV disease with associated ascending aortic aneurysms. 

## 2. Prevalence

It is commonly accepted that bicuspid aortic valve disease has a prevalence of 1 to 2% in the general population with between a 2 : 1 and 4 : 1 predilection for males : females [1–10] (Table 1). In the largest necropsy study to date, 21 000 individuals were examined and bicuspid aortic valves were present in 569 (1.4%) [2]. However, necropsy studies may underestimate the true prevalence due to selection and misclassification bias. More recently, in a screening transthoracic echocardiography study of 1075 newborns, the incidence of BAV was determined to be 4.6 in every 1000 live births [4], with a 4 : 1 male : female ratio. 

## 3. Anatomy of the BAV

### 3.1. Embryology

 The exact cause and mechanism responsible for the development of the BAV is uncertain. The hemodynamic moulding theory suggests that decreased blood flow through the valve during development resulted in a failure of the leaflets to separate; however, there is limited evidence to support this theory. Another popular explanation has been a genetic abnormality, though current studies have been unable to consistently determine specific genetic defects associated with BAV disease. 

 Fernández and associates [10] studied aortic valve development in inbred Syrian hamsters and endothelium nitric oxide synthase (eNOS^−/−^) knockout mice, both of which have a high prevalence of BAV [11, 12]. Using histological sections, this group was able to show that the most common BAV morphologies were a result of two separate developmental defects. Fusion of the left- and right-coronary cusps in the Syrian hamsters was a result of extra fusion of the septal and parietal ridges and subsequent defective outflow tract septation. The posterior cushion developed normally and became the posterior cusp, as in the normal AV. Formation of an BAV in the eNOS^−/−^ mice was a result of fusion of the posterior intercalated cushion with the septal ridge followed by normal outflow tract septation, leading to fusion of the right and noncoronary cusps. These novel findings demonstrated that, at least in animal models, different BAV morphologies are the result of different developmental processes. If this process is similar in humans, it may help to explain the heterogeneous nature of BAV disease.

 More recently, Sans-Coma and colleagues [13] were able to demonstrate that a continuum of aortic valve morphologies, ranging from normal tricuspid valves to pure bicuspid valves, could develop in genetically alike Syrian hamsters. This finding suggests that factors other than genetics may play a role in the development of the BAV. 

### 3.2. Classification of BAV

**Figure 1:**
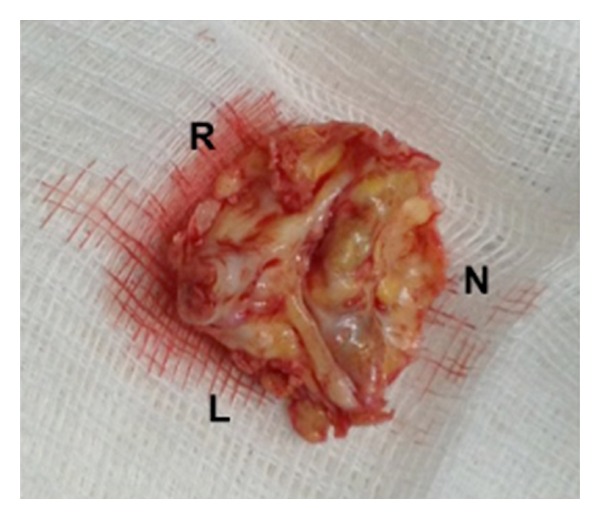
Photograph of surgically excised bicuspid aortic valve, demonstrating severe calcific stenosis. The left (L) and right (R) cusps are fused with a prominent calcified raphe, opposed to a calcified noncoronary cusp (N).

The bicuspid aortic valve is often identified by an abnormally large aortic valve cusp with a prominent raphe in an area of cuspal fusion. Fusion of the left-coronary and right-coronary cusps is the most common morphology reported in over 60% of BAV cases (Figure 1). Fusion of the right-coronary and noncoronary cusps occurs in 15–25% of cases, while fusion of the left-coronary and noncoronary cusps is quite rare, occurring in less than 5% of individuals with BAV [14–19]. The interleaflet triangle between the two fused cusps is usually much smaller than normal and can lead to decreased mobility of the cusp [20]. Abnormal creasing of the anomalous cusp during the cardiac cycle is also common [21]. 

 Three major classification schemes of BAV disease have been described to create a common language for diagnosis, prognosis, and surgical planning. Sievers and Schmidtke [22] described a detailed anatomic classification in 2007 based on pathologic examination. According to this nosology, BAV has two functional cusps forming the valve mechanism with less than three zones of parallel apposition between cusps. The valves are categorized according to the number of raphes present (e.g., 0, 1, or 2) and by the spatial orientation of the cusps and location of the raphe(s). The valves are further subcategorized by function-normal, insufficient, stenotic, or “balanced” (both moderate stenosis and insufficiency). The most common type of BAV by the Sievers' classification is type 1, L-R, S, indicating one raphe between the left- and right-coronary cusps with a hemodynamically predominance of stenosis (Figure 1).

 Recognizing associated aortic dilatation in BAV disease, Schaefer and colleagues [18] proposed an integrated classification system based on both cusp fusion and root shape. The cusp fusion classification system is similar to other groups; however, they also described three distinct aortic root shapes termed type N, type A, and type E. In type N “normal” roots, the sinus diameter is greater than that of the sinotubular junction diameter and greater than or equal to the ascending aortic diameter. In Type A “ascending dilatation” roots, the sinus diameter is greater than that of the STJ diameter and less than the diameter of the ascending aorta. Finally in Type E “effaced” roots, the diameter at the level of the aortic sinuses is equal to or less than the diameter of the STJ. In this series, patients with fusion of the L-R coronary cusps, most commonly presented with Type N root anatomy while those with fusion of the right-coronary and noncoronary cusps were more likely to have a Type A root anatomy. The Type E root anatomy was found in 5% of patients with L-R cusp fusion and 14% of patients with R-N cusp fusion. 

**Figure 2:**
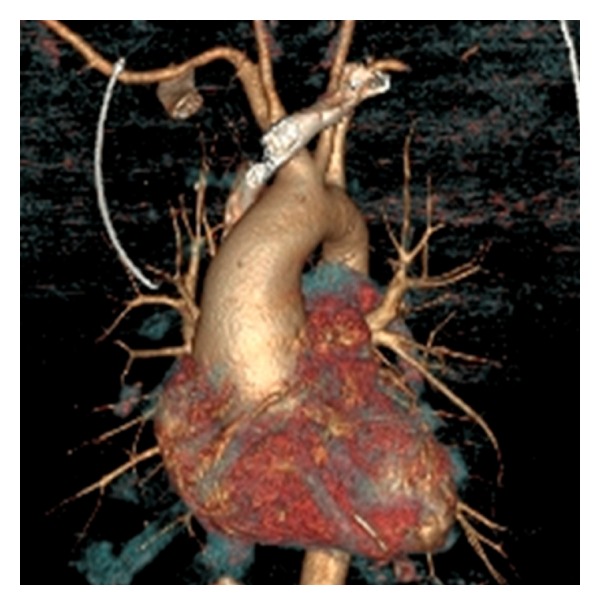
Computed tomography of a patient with a bicuspid aortic valve and aneurysmal dilatation of the aortic root, ascending aorta, and transverse aortic arch (Stanford cluster IV).

 Fazel and coworkers [23] from Stanford, CA, USA analyzed 64 BAV patients and described four distinct patterns of aneurysmal aortic involvement (Table 2). Cluster I involved aortic root dilatation alone, cluster II involved dilatation of the tubular ascending aorta alone, cluster III involved dilatation of the tubular ascending aorta and aortic arch, and cluster IV involved dilatation of the aortic root, tubular ascending aorta with tapering across the transverse arch. Seventy-three percent of the patients had involvement of the aortic arch (clusters III and IV) (Figure 2). Recently, two new clusters Ia (STJ-preserved) and Ib (STJ-dilated) have been reported [24]. These distinct patterns of aortic involvement reinforce the need for an individualized, custom-tailored degree of ascending aortic and arch replacement in patients with bicuspid aortopathy.

### 3.3. Pathophysiology

 BAV disease can present with various hemodynamic derangements including stenosis, insufficiency, and mixed presentations. The predominant hemodynamic lesion in BAV disease may be related to the age of presentation, cuspal fusion patterns, and flow dynamics. In the elderly population, aortic stenosis is the most common presentation affecting 75% patients undergoing surgery for BAV disease, while insufficiency is the reason for intervention in only 13–16% of BAV patients [15, 25]. In infancy, aortic stenosis is much more common than insufficiency in BAV disease where approximately 80–95% of cases of aortic stenosis detected in early life can be attributed to a bicuspid or unicuspid valve [26, 27]. 

There has been some suggestion that certain BAV morphologies are more highly correlated to the presence of either a stenotic or regurgitant lesion. In a study of 569 pediatric patients with isolated BAV, significant aortic stenosis was more than twice as likely in patients with fusion of the right-coronary and noncoronary cusps, whereas fusion of the left-coronary and noncoronary cusps had no association with aortic stenosis [14]. Fusion of the right-coronary and noncoronary cusps also had a twofold higher odds of having at least moderate aortic regurgitation. This may suggest that right-coronary and noncoronary cusp fusion may have worse long-term prognosis because of more hemodynamically significant lesions. Interestingly, these morphological correlations to specific lesions seem to disappear in the adult population [16, 17] perhaps because of their earlier age of presentation. In adults with BAV, progression of AS appears to progress more rapidly in patients with cusps in the anteroposterior position or left- and right-coronary cusp fusion [28].

In the normally functioning tricuspid aortic valve, the aortic cusps are relatively similar in size, opening into their respected sinuses during systole, and coapting equally during diastole to equalize pressure dynamics across the aortic root. In the BAV, aortic cusps often do not fully open [21] and coaptation is often eccentric [29]. Together, these abnormalities can produce an elliptical orifice area and flow turbulence, perhaps predisposing to early valve degeneration and calcification leading to clinically significant AS (Figure 1), up to a decade earlier than individuals with tricuspid aortic valves [30]. Echocardiographic studies have shown that in BAV patients, cuspal sclerosis typically begins in the second decade of life while calcification is prominent in most middle-aged patients [28]. This early degeneration may be related to more aggressive inflammatory changes of the aortic valve, characterized by increased macrophage infiltration and neovascularization [31]. 

**Figure 3:**
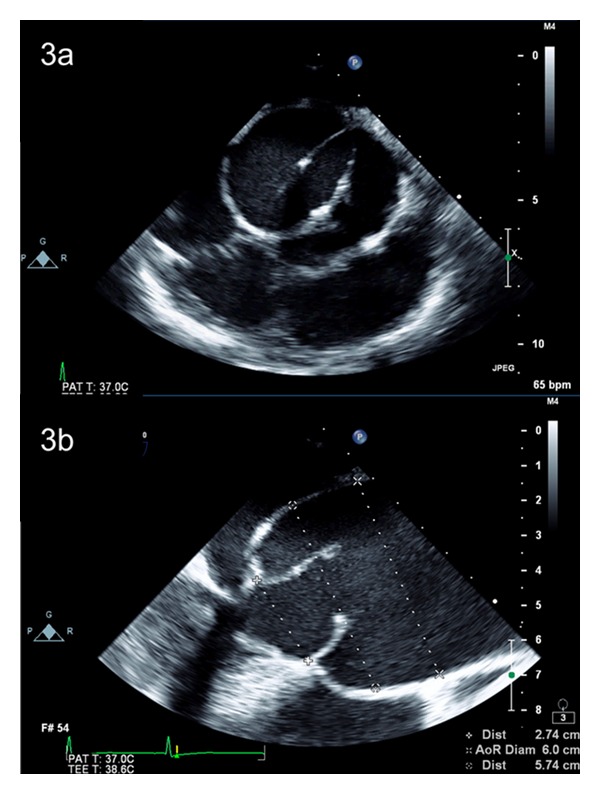
(a) Transesophageal echocardiogram demonstrating a bicuspid aortic valve in short-axis view, with left-right coronary cuspal fusion (Sievers' classification type 1, L-R, insufficient), and moderate-severe aortic insufficiency on colour flow Doppler (not shown). (b) Transesophageal echocardiogram demonstrating a dilated aortic root and ascending aorta in long-axis view along with a bicuspid aortic valve.

 Aortic insufficiency in BAV disease is often mild to moderate in severity and concurrent with aortic stenosis, although predominant insufficiency can occur. Development of AI can be attributed to several different characteristics of the BAV. Firstly, as a result of differences in leaflet dimensions, 15–20% of all BAVs have incomplete closure [32]. Redundancy in the fused leaflet also predisposes the BAV to cuspal prolapse leading to the onset of AI [33] (Figure 3(a)). Furthermore, dilatation of the aortic root and sinotubular junction are common traits of BAV disease (Figure 3(b)). This dilatation is often progressive and can lead to deterioration of valvular function. Studies have shown a 36% decrease in coaptation height and a 41% decrease in contact pressure [29] between leaflets in BAV, both of which are likely to be further exacerbated by dilatation of the aortic root and sinotubular junction. Isolated severe insufficiency is relatively uncommon in the setting of BAV and when present, is often related to infective endocarditis [34, 35].

### 3.4. Associated Abnormalities

 Bicuspid aortic valves do not always present in isolation and are commonly associated with other congenital cardiovascular defects. The most robust association occurs with coarctation of the aorta, where up to 3/4 of individuals with aortic coarctation also have coexistent BAV [36, 37]. In this specific BAV population, there appears to be a preponderance of morphological fusion of the left-coronary and right-coronary cusps [14, 19]. Bicuspid aortic valves are also more commonly linked to other left-sided obstructive lesions including interrupted aortic arch [38], Shone's complex [39], and hypoplastic left heart syndrome [40–42]. Other congenital lesions associated with BAV include patent ductus arteriosus, ventricular septal defect, and atrial septal defects. Several studies have also, noted variations in the coronary anatomy in patients with BAV with an increased prevalence of left dominant coronary circulation and shorter left main coronary arteries [43–46].

## 4. BAV and Aortic Aneurysms

**Figure 4:**
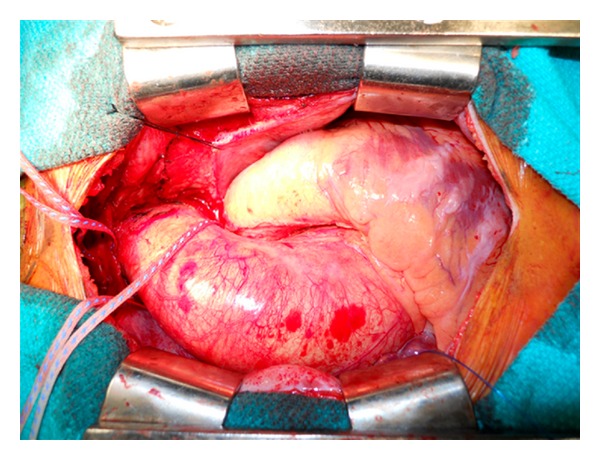
Intraoperative photograph demonstrating the dilated aortic root, ascending aorta, and proximal aortic arch (Stanford cluster IV), measuring 68 mm in the largest dimension on preoperative computed tomography (not shown).

 Recently, there has been increasing recognition of the association between BAV and ascending aortic dilatation (Figures 2 and 4). Abbott was the first to suggest a link between bicuspid aortic valves and ascending aortic aneurysms in 1928 [47] and indeed one of the most consistent findings in BAV is dilation of the ascending aorta, even in the absence of clinically significant valvular dysfunction [48, 49]. Dilatation of the ascending aorta represents a key risk factor for dissection and rupture, both of which are major causes of morbidity and mortality. Some of the earliest observational studies suggested a ninefold increased risk of aortic dissection in these individuals [2, 50, 51]. However, the generalizability of these data is limited by the fact that it was based on necropsy findings. More recent investigations suggest that while the risk of dissection in patients with BAV disease is higher than the general population, it is lower than originally postulated [52, 53]. Dilatation of the aortic root and proximal ascending aorta is one of the most common nonvalvular finding in patients with BAV disease with an incidence between 30 and 70% [54–57]. It appears that the morphology of the BAV may also be predictive of the location and type of dilatation of the aorta. Compared to patients with fusion of the R-N cusps, patients with fusion of the L-R cusps typically present with larger annular and sinus dimensions and smaller arch diameters. The diameter at the level of the STJ and ascending aorta is similar in both morphologies [17, 18].

## 5. Pathophysiology of Aneurysmal Dilatation in BAV

### 5.1. Hemodynamic Theory

 Hemodynamic derangements of the BAV, including abnormal flow turbulence, poststenotic dilatation, and increased stroke volumes of aortic insufficiency [21, 55, 58, 59], are believed to be the most common cause for aneurysmal dilatation of the aortic root and ascending aorta. As a result, aortic dilatation begets more dilatation because of the larger aortic diameter, decreased wall thickness, and overall increased wall tension (Laplace's law), which can ultimately result in catastrophic aortic rupture, dissection, or death. Support for this theory was provided by several studies which demonstrated an association between significant valvular disease in BAV and aortic root size [58, 59]. However, other investigations have suggested that even patients without significant valvular dysfunction have larger aortic diameters compared to those with tricuspid AV [48, 49, 60]. While in the past, some have used these findings to suggest that hemodynamic derangements are not the only cause of ascending aortic aneurysms in patients with BAV, recent advances in medical imaging techniques have allowed for further investigation of the flow patterns in patients with bicuspid aortic valve disease. 

In a very interesting study by Hope et al. [61] using 4D flow MR imaging, distinct patterns of abnormal flow were identified in patients with BAV even in the setting of a “functionally normal” bicuspid valve. These findings support the earlier work by Robicsek and colleagues [21] who determined that the clinically normal bicuspid aortic valve is in fact morphologically stenotic and produces eccentric turbulent transvalvular flow. It is possible then that these eccentric flow patterns may in turn lead to a differential distribution of aortic wall shear stress and subsequent flow-induced vascular remodeling of the aortic wall [17]. The group of Conti and associates [29] found a 36% increase in longitudinal wall stress at the greater curvature of the ascending aorta in patients with BAV disease compared to those with tricuspid AV. More recently, Vergara and associates [62] demonstrated similar differences in wall stresses and flow in patients with BAV disease. These changes seem to corroborate the greater degree of extracellular matrix disarray, smooth muscle cell changes, and asymmetric dilatation noted in patients with BAV [63–65]. There also appears to be an increase in vascular smooth muscle cell apoptosis in this particular region of the aorta [64, 66, 67]. 

### 5.2. Aortopathy

#### 5.2.1. Medial Degeneration

 Histological abnormalities of the aortic media in patients with bicuspid aortic valve disease are well documented [68]. Studies have demonstrated that the aortic media above a bicuspid aortic valve are abnormal regardless of valve function [69] and are also present in the pulmonary trunk [70], lending support to the presence of an underlying systemic disorder. 

 Many different groups have confirmed the presence of cystic medial necrosis (CMN) in patients with BAV disease, which is characterized by vascular smooth muscle cell (VSMC) loss in the absence of inflammation, elastic fiber fragmentation, and accumulation of basophilic ground substance within cell-depleted areas of the ascending aortic media [70, 71]. Importantly, cystic medial necrosis is the underlying histological abnormality in ascending aortic dilatation and dissection [72, 73]. 

 High rates of VSMC apoptosis and medial degeneration are present even in nondilated ascending aortas [72, 74], which suggests an underlying abnormality in patients with BAV. The convexity of the aorta is especially prone to high rates of VSMC apoptosis [64, 66, 67] as well as medial degeneration [75] which may explain the higher incidence of aortic dilatation in this region [29]. VSMC may play a crucial role in remodeling of the aortic media by producing extracellular matrix proteins including collagen, elastin, and fibrillin [66, 67]. Nataatmadja and colleagues [66] demonstrated defective protein transport from VSMC to the extracellular matrix leading to intracellular accumulation of fibrillin, fibronectin, and tenascin in VSMC and decreased extracellular deposition of fibrillin. This defect in protein transport may play a role in the development of aortic aneurysms by resulting in improper maintenance of the extracellular matrix and VSMC apoptosis. This group proposes that the loss of VSMC is the primary cause of aortic wall weakness in patients with Marfan's syndrome. 

 The histological changes seen in BAV appear to be part of a continuum of aortopathy with aneurysms in tricuspid aortic valves and Marfan's patients representing the extremes. The histological changes in BAV are similar, though less severe than those found in patients with Marfan's syndrome [72]; however, they appear to be more severe and occur at an early age than in patients with tricuspid AV [67]. 

#### 5.2.2. Abnormal Fibrillin

 Fibrillin-1 is a crucial component of extracellular matrix that forms microfibrils with other extracellular matrix molecules such as elastin [76]. These fibrillin-rich microfibrils play a crucial role in maintaining tissue elasticity by anchoring vascular smooth muscle cells to elastin and collagen [77]. Abnormalities in the amount of fibrillin-1 have been found in the aortic valve, aorta, and pulmonary valve in patients with congenitally bicuspid aortic valves [70]. Deficiencies of fibrillin-1 result in VSMC detachment, matrix disruption, and apoptosis [78] and ultimately results in a fragile aorta, less suited to deal with stress associated with valvular dysfunction. Fedak et al. also [79] found decreased levels of fibrillin-1 in both the ascending aorta and the pulmonary trunk in patients undergoing aortic surgery. Though this finding suggests that the fibrillin deficiency may be a systemic issue, it is not possible to determine whether it is the result of a primary genetic defect. Mutations of the FBN1 gene, which encodes fibrillin-1, are associated with the development of Marfan's syndrome. Patients with Marfan's syndrome also commonly develop ascending aortic aneurysms and have a higher than normal prevalence of BAV.

#### 5.2.3. Increased MMP Activity

 Matrix metalloproteinases (MMPs) are a large family of zinc-dependant endopeptidases responsible for degradation of extracellular matrix. There is currently a growing body of evidence implicating these MMPs in ascending aortic aneurysm formation. To date, most studies have focused on MMP-2 and MMP-9 which belong to a subclass of MMPs known as the gelatinases. MMP-9 has been closely associated with the formation of abdominal aortic aneurysms [80, 81] and more recently with dilatation of the ascending aorta. Several studies have found increased levels of MMP-9 in the aneurysmal ascending aortas in patients with BAV [82, 83], while others have found a significantly increased expression in aneurysmal aortas in patients with tricuspid AV compared to patients with BAV [84]. This seemingly contradictory data may be a result of the exclusion of patients with comorbidities such as atherosclerosis in the former two investigations. The published data, although not definitive, are more consistent with regard to levels of MMP-2 in aneurysmal ascending aortas. Increased MMP-2 has been demonstrated in patients with BAV compared to both control groups and tricuspid AV groups [79, 84–87]. Increased turbulence, present even in normally functioning BAV [61], has been shown to lead to MMP-2 activation [88].

In the aortic media, MMP activity is regulated by the presence of tissue inhibitors of matrix metalloproteinases (TIMPs). It is no surprise then that increases in the MMP : TIMP ratio may also play a role in aneurysm formation in individuals with BAV disease. In a study of surgically excised aortic valves, Wilton and colleagues [89] were unable to find differences in the level of expression of MMPs and TIMPs; however, they did find that there was a significant difference in the ratio of MMP-2 to TIMP-1 in patients with BAV compared to those with a tricuspid AV. Similarly, Lemaire and associates found a significant increase in MMP-2/TIMP-2 ratio in BAV aneurysms compared to control aortas. A recent and very interesting investigation by the group of Ikonomidis et al. [90] discovered that each BAV morphology has a unique pattern of MMP and TIMP activity. Their investigation revealed that individuals with L-R morphology have an elevated MMP/TIMP score ratio, suggesting that extracellular matrix degradation in these patients may be more aggressive.

Unfortunately, many of the investigations were limited by relatively small sample sizes and the inability to distinguish whether or not abnormalities in MMP and TIMP activity were causative or a result of aortic aneurysm development. 

#### 5.2.4. Genetics

 Familial links have been identified in BAV disease and suggest an autosomal dominant inheritance pattern with reduced penetrance [96, 97]. BAV with concomitant ascending aortic dilatation also appears to be transmitted with a similar inheritance pattern and is associated with a spectrum of left-sided obstructive lesions [98]. Unlike Marfan's and Loeys-Dietz syndromes, both of which can involve BAV and dilatation of the ascending aorta, no causative gene for BAV disease has been identified. Recently, it was discovered that a small number of patients with BAV disease both with and without dilatation of the aorta possess mutations of the NOTCH1 gene [99, 100]. The involvement of NOTCH1 is a particularly interesting finding as NOTCH1 also plays a role in guiding neural crest migration during valvulogenesis. Mutations of the NOTCH1 gene leading to abnormalities in neural crest migration would explain the involvement of the aortic root and ascending aorta in BAV disease, as they are of the same neural crest cell derivatives. As a result, surgical resection of the entire proximal aorta has been rationalized in some patients with BAV and ascending aortic aneurysms.

 Another potential argument for the genetic theory for aneurysm formation in the setting of BAV is the observed late dilatation of the pulmonary autograft after the Ross procedure. Since the aortic valve, proximal ascending aorta, and pulmonary trunk are all derived from the same neural crest cell lines [101], this could again potentially implicate a genetic etiology. However, contrary to patients with Marfan's syndrome, reports have shown that dilatation of the pulmonary trunk in situ is quite rare in patients with BAV disease [101]. Adding further to the genetic argument, Martin et al. [102] recently demonstrated that the aorta and pulmonary artery are significantly larger in patients with BAV and these measurements are traits that exhibit significant heritability. 

## 6. Aneurysm Growth

### 6.1. Rate of Growth

 The rate of growth of the ascending aorta in patients with BAV is slow, ranging from 0.2 to 1.9 mm per year [52, 56, 103–105]. In accordance with Laplace's Law, larger aortas have faster expansion rates [106–108]. In one study, aortas with an initial diameter of 35 mm to 40 mm had an expansion rate of 2.1 mm/year, whereas aortic aneurysms of 6 cm or larger had expansion rates of 5.6 mm/year [107]. Some individuals in the aforementioned studies showed either no growth or a decrease in aortic diameter with time, exemplifying that estimation of aortic dilatation is difficult due to variability in image readings [109], short follow-up periods, small sample sizes, and selection bias [72]. Acute aortic dissection should be suspected when the thoracic aorta enlarges rapidly in a short period of time [110]. 

### 6.2. Location of Growth

 Dilatation of the aorta in BAV disease most commonly occurs in the ascending aortic segment; however, dilatation can occur anywhere between the aortic root and the aortic isthmus [111]. This pattern of aortic dilatation is thought to be related to the embryonic derivation of these structures from the same neural crest derivatives [70, 78, 112]. As previously mentioned, clusters of thoracic aortic aneurysm morphology have been identified in patients with BAV (Table 2).

### 6.3. Absolute Size Criteria versus Relative Sizes/Ratios

 In general, recent guidelines have recommended surgery for patients with BAV and ascending aortic aneurysms of greater than 50 mm in diameter [113]. Absolute size measurements should be carefully acquired by computed tomography or magnetic resonance imaging, within the axial plane of the aorta to avoid overestimating aortic diameters. Mendoza et al. suggest that using aortic size as determined from double oblique (DO) plane on CT is the most accurate method of determining when patients meet the size criteria for aortic surgery [114]. Echocardiography often measures the inner diameter of the proximal and distal aorta and tends to underestimate the actual aortic dimensions or misses the largest extent of the distal ascending aorta all together. Although not definitively validated, aortic size ratios and indexes should be considered for adults with BAV and small body size [71]. Elective aortic resection has been advocated for BAV patients with aortic diameters >45 mm and either of the following (Table 3):

1. ratio of aortic area to body height >10 cm^2^/m in asymptomatic patients with well-functioning BAV, or 8-9 cm^2^/m in symptomatic patients [115];
2. ratio of aortic diameter to body surface area >45 mm/m^2^ [116].

Higher ratios indicate >20% annual risk of aortic dissection, rupture, and death.

Ergin et al. [91] also suggest employing age/body size-adjusted formulae for determining the predicted aortic dimensions at the level of the sinuses. This group recommends intervention on the aorta when the ratio of measured diameter: predicted diameter is 1.4 or greater, in patients with BAV. 

## 7. Natural History of BAV and Ascending Aortic Aneurysms

 Aortic diameter appears to be a significant predictor of aortic dissection, aortic rupture, and aorta-related death. From a database of the International Registry of Aortic Dissection of 1600 thoracic aortic aneurysms and dissections, aortas >6 cm had annual rates of rupture, dissection, and aorta-related death of 3.6%, 3.7%, and 10.8%, respectively [92]. The cumulative rate of any of those events was 14.1%, more than double the rate of adverse events for aortic aneurysms between 5 and 6 cm (6.5%). BAV-associated ascending aortic aneurysms dissect and rupture at a size range comparable to that of aneurysms due to other etiologies [93, 115]. The increased risk of rupture associated with BAV is due to a higher prevalence and rate of aortic dilatation, which occurs at a significantly younger age relative to idiopathic ascending aortic aneurysms [30, 94, 103]. However, patients with BAV clearly consist of a heterogeneous group, and diameter and rate of growth alone are not the only factors contributing to the increased risk of rupture. As we have previously discussed, many patients with BAV likely possess tissue, genetic, and molecular abnormalities which may contribute to the increased risk of aneurysmal rupture and dissection in patients with normal aortic dimensions. 

 Although original reports suggested that BAV disease carries a 6.14% lifetime risk of aortic dissection, 9-fold higher than the risk in the general population [94], more recent investigations indicate dissection rates to be generally low. In a community-based study, Michelena and colleagues [52] followed 416 consecutive patients with confirmed BAV. Two of 416 patients experienced aortic dissections during a mean followup of 16 ± 7 years (3.1 cases per 10 000 patient-years). One of the patients who experienced aortic dissection had a previous AVR, the other patient had moderate AS. At the time of BAV diagnosis, 32 patients met the criteria for aortic aneurysm (diameter >45 mm) and subsequently underwent aortic surgery during followup (15 ± 6 years). Of the 384 patients without aortic aneurysm at the time of diagnosis, 49 developed aortic aneurysms and almost half of these patients underwent elective aortic surgery. No dissection occurred in individuals without an aortic aneurysm at the time of diagnosis. Tzemos and colleagues [53] have previously reported similar results in a series of 643 patients with confirmed BAV disease followed for an average of 9 years. During followup, 142 (22%) required ascending aorta or aortic valve intervention. Eleven of these patients underwent intervention as a result of dilatation of the ascending aorta. Aortic dissection (3 ascending, 2 descending) occurred in five patients (0.77%), two of which resulted in death (1 preoperative, 1 postoperative). The overall frequency of dissection was 0.1% per patient-year of followup. 

 Although dissection rates in the current era are lower than previously believed, they remain significantly higher than in the general population. Consistent clinical followup remains crucial in patients with BAV disease as approximately 10% of patients undergoing clinical surveillance for a normally functioning BAV and aortic aneurysm will require surgical intervention each year [95].

**Fate of the Ascending Aorta after AVR**

Persistent dilatation of the ascending aorta in BAV disease due to hemodynamic derangements should theoretically be relieved by AVR; however, the evidence remains controversial. Published long-term data evaluating aortic events after AVR range from quite adverse to seemingly benign [30, 53, 57, 117–121]. Borger and coworkers [118] evaluated 201 patients with BAV who underwent AVR for an average of 10.3 ± 3.8 years. They found a low prevalence of both subsequent aortic dissection/rupture (0.5%) and sudden cardiac death (1.5%); however, 18 (9%) patients required intervention on the ascending aorta. A significant proportion of the individuals undergoing aortic surgery also required concomitant AVR as a result of structural valve deterioration, hence confounding the primary determinant of reoperation. A very recent investigation by Girdauskas et al. [119] demonstrated similar and encouraging results in patients with BAV, AS, and mild-to-moderate dilatation (40–50 mm) of the ascending aorta. Freedom from aortic intervention was 97% and 94% at 10 and 15 years, respectively. No cases of aortic dissection or rupture were document and ascending aortic surgery was required in only five patients (3%) for progressive ascending aortic aneurysm. Furthermore, this group found that in a subgroup of patients with aortic insufficiency (*n* = 21), the freedom from adverse aortic events was significantly higher (*P* = 0.009) with 24% of patients experiencing an adverse event, including aortic root aneurysm, acute type A dissection, and sudden cardiac death. This finding is similar to those of Yasuda and colleagues [120] who showed that progression of aortic dilatation was greater (although not statistically significant) in patients who underwent AVR for BAV and associated AI. In this investigation; however, data showed that all patients with BAV, regardless of operative status, showed progressive dilatation of the aorta over time. Unfortunately, this study had a very small patient population and excluded patients with dilatation of the ascending aorta (>44 mm) at the time of intervention.

Perhaps the most worrisome data regarding the fate of the ascending aorta after AVR was presented by Russo et al. [121]. They followed 50 patients for an average of 19.5 ± 3.9 years after AVR and found high rates of rupture (10%), aortic reoperations (6.0%), and sudden deaths (14%), suggesting that an underlying condition was implicated in the formation of aortic aneurysms in patients with BAV disease. In balance, it is evident that significant conflicting evidence exists, as we incompletely understand this heterogeneous disease and aortic events after AVR for BAV disease cannot be clearly predicted.

## 8. Nonoperative Management

### 8.1. *β*-Blockers

 Halpern et al. were the first to suggest the efficacy of *β*-adrenergic blockers in slowing the dilatation of the ascending aorta as a result of observations in a small group of patients [122]. This preliminary report lead to the landmark study by Shores et al. published in 1994 [123]. In this randomized trial of predominantly adolescent participants, the patients treated with an individualized dose of propranolol experienced aortic dilatation rates, one-third of those patients in the control group. A significantly lower incidence of clinical endpoints (16% versus 24%) was also noted between experimental and control groups. Similar results with respect to decreased rates of aortic dilatation with *β*-blocker therapy have been confirmed by others, with trends towards lower cardiac mortality and fewer aortic dissections [124]. Generalizability of these studies is limited by the small sample sizes of each trial and the fact that all patients had Marfan's syndrome. In contrast, a retrospective investigation by Selamet Tierney and associates [125] found no difference in the rate of aortic dilatation in patients receiving *β*-blocker therapy compared to a control group. The role of *β*-blocker therapy in the management of BAV aortopathy has yet to be established. 

### 8.2. Angiotensin Receptor Blockers

 Angiotensin receptor blockers (ARBs) have also been identified as potential therapeutic agents to combat progressive dilatation of the ascending aorta. Experimental mouse models with mutations of the FBN-1 gene, treated with pre- or post-natal losartan, showed no difference in aortic diameters compared to their wild-type littermates. Furthermore, elastic fragmentation was also prevented by administration of losartan [126]. The slowed progression of aneurysmal growth appears to be a result of attenuation of TGF-*β* signaling in the aortic media. Losartan has also been investigated in non-Marfan animal models prone to aneurysmal disease. In these animals, angiotensin 1 (AT1) receptor antagonists reduced haemodynamic stress and improved lifespan; however, the aortic media structure was unaffected. 

 Due to the efficacy of ARB treatment in animal models, there is hope that losartan therapy may also attenuate dilatation of the ascending aorta in human Marfan's patients. There are currently two ongoing clinical trials investigating the efficacy of ARB therapy: the COMPARE trial [127] in The Netherlands and Marfan Sartan trial in France [128]. There is also an important ongoing Canadian trial that is currently enrolling BAV patients (BAV Study) and randomizing them to long-term *β*-blocker therapy (atenolol) and/or ARB (telmisartan) to assess their efficacy to reduce aortic dilatation from baseline [129]. These study results will hopefully provide much needed insight into the utility of *β*-blocker or ARB treatment to reduce aortic dilatation and hopefully aortic events in patients with BAV.

## 9. Operative Management

 Surgical management of BAV disease with concomitant ascending aortic aneurysm has often been treated with a straightforward approach that addresses each problem individually. However, because of the heterogeneous presentation of BAV disease and the gaps in knowledge of the associated aneurysmal behavior and molecular characteristics, a thoughtful approach carefully assessing individual risk factors of the aortic valve and aorta is required to determine the most appropriate surgical intervention for optimal outcomes. Current guidelines of the European Society of Cardiology (ESC) [130] and the joint guidelines of the American College of Cardiology (ACC)/American Heart association (AHA) [113] recommend elective aortic repair in patients with a proximal aortic diameter >45 mm and concomitant indication for elective aortic valve repair/replacement. In asymptomatic patients with well-functioning BAV, elective repair is recommended for diameters ≥50 mm, if aneurysmal dilatation is >5 mm/year, if the patient has a strong family history of dissection/rupture/sudden death, or if pregnancy is planned.

**Figure 5:**
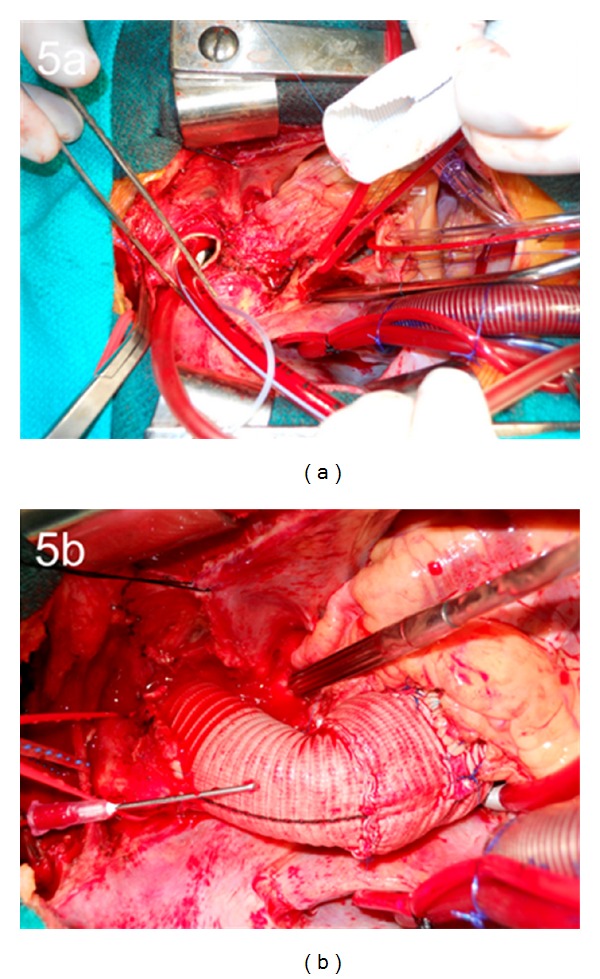
(a) Intraoperative photograph demonstrating the aortic resection from the aortic valve to the aortic arch, utilizing the Peninsula technique. (b) Intraoperative photograph demonstrating the reconstructed aortic root, ascending aorta, and aortic arch.

 Judgment calls are often required to determine how aggressive of a surgical strategy towards valve repair versus replacement and how much aortic resection is necessary to prevent late aneurysm recurrence. When the ascending aorta is significantly dilated (>50 mm diameter), ascending aortic replacement with a tube graft is commonly performed. However, the challenging decision making often lies at the proximal and distal ends of the aortic resection. Considering the molecular and genetic research identifying abnormal aortic wall tissue in BAV disease, do these abnormalities manifest late complications in the aortic root or aortic arch and should these segments of aorta be left behind following aortic valve and ascending aorta replacement? The clinical evidence that we presented to date is contradictory; however, this must be interpreted with caution in the setting of the mounting genetic and histologic evidence supporting a more diffuse process affecting at least the proximal aortic segments. Practically, how aggressive should a surgical strategy be if the proximal and distal aortic ends are dilated but do not reach conventional criteria for operative resection (i.e., aortic root, ascending aorta, and aortic arch measure 40 mm, 55 mm, and 40 mm, resp.)? The surgeon must carefully weigh the theoretical and perhaps uncertain long-term benefits of more aggressive aortic resections versus the increased perioperative risks of additional aortic root and aortic arch resections (Figures 5(a) and 5(b)). 

 Since very little evidence exists to guide these operative decisions, we advocate for an individualized approach tailoring the surgical procedure to provide the lowest perioperative risk with the optimal long-term outcome. More aggressive aortic resections are considered when patients have worrisome negative prognostic risk factors. These include history of connective tissue disorders or other arterial aneurysms, family history of aneurysms or aortic catastrophe, rapid progression in aortic dilatation, associated cardiovascular abnormalities, or significant aortic wall thinning or fragility when identified intraoperatively. 

 As most patients with BAV disease and ascending aortic aneurysms present with significant calcified aortic valve stenosis, aortic valve replacement is commonly required. Both AVR plus ascending aorta replacement and composite aortic root replacement can be performed with excellent outcomes [131–133]. Zehr et al. [132] demonstrated excellent outcomes in a series of 206 BAV patients undergoing the modified Bentall procedure. Patients experienced low operative mortality (2.9%), and no patients required reoperation of the aortic during followup (mean 5.9 years). Furthermore, these patients also have life expectancies similar to those of an age/sex matched population, leading some to suggest that the modified Bentall may be an optimal surgical procedure in patients with BAV. The modified Bentall may be an especially attractive option when the surgeon has little experience with valve-sparing techniques or when the valve is not suitable for repair. 

**Figure 6:**
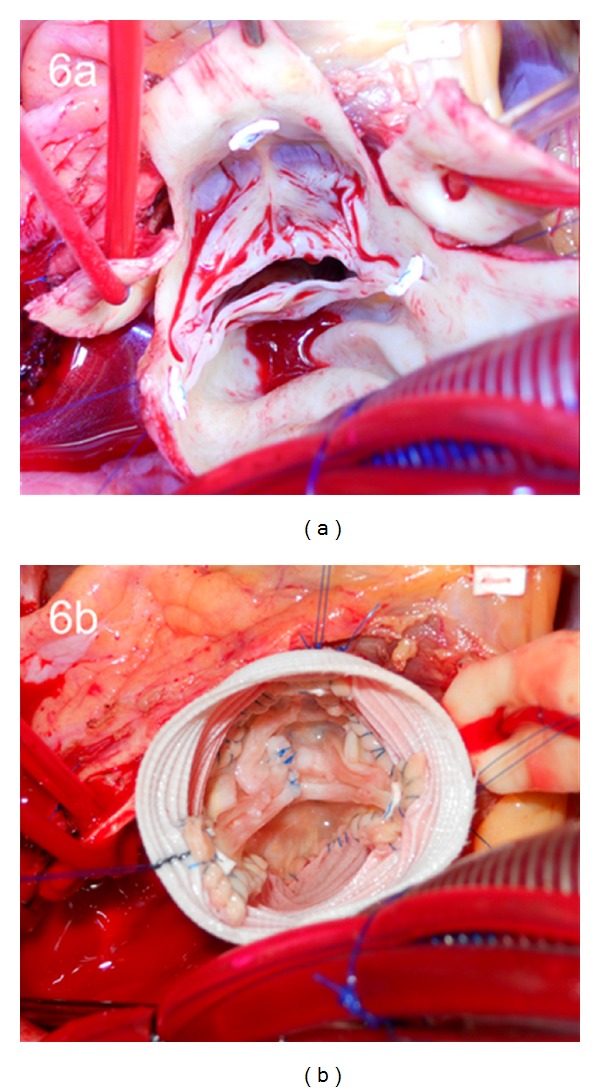
(a) Intraoperative photograph demonstrating the bicuspid aortic valve in situ with the raphe between the left- and right-coronary cusps. (b) Intraoperative photograph demonstrating the results of the valve sparing aortic root replacement using the reimplantation technique. The free margin of the conjoined cusp was plicated and the free margin of the nonconjoined cusp underwent a triangular resection.

**Figure 7:**
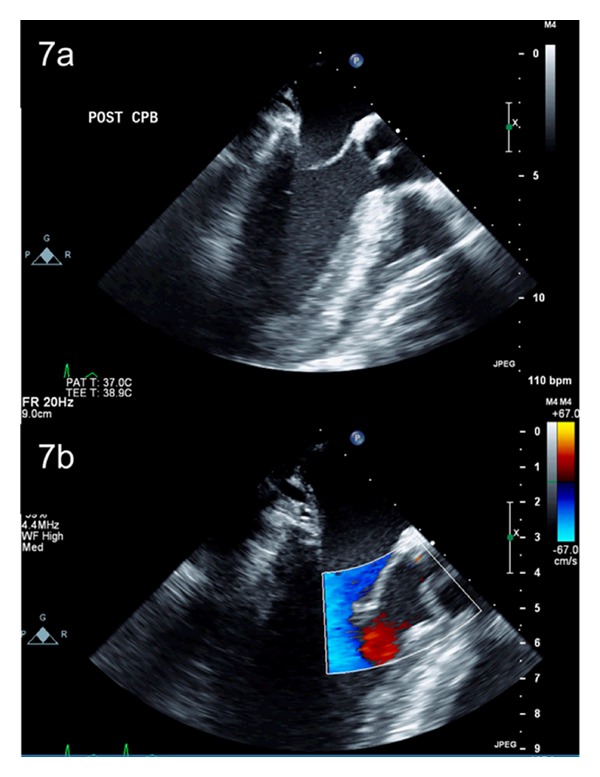
Postoperative transesophageal echocardiogram demonstrating good coaptation of the repaired aortic valve (a), without any residual aortic insufficiency on colour flow Doppler (b).

 Aortic valve replacement relieves symptoms and improves survival; however, it exposes patients to prosthesis-related complications which may be more relevant in BAV patients who tend to be younger at time of surgical intervention. As a result, patients with noncalcified, mobile, and predominantly insufficient bicuspid aortic valve cusps with cuspal orientation near 180° should be considered for aortic valve sparing procedures. Aortic valve reimplantation and aortic root remodeling techniques allow for native valve preservation while simultaneously treating the aortic root aneurysm (Figures 6(a), 6(b), and 7) and have been performed with excellent results [134–138]. These aortic valve repair procedures for BAV have been most successful when the underlying insufficient BAV is a result of annular dilatation and/or cuspal prolapse, rather than restrictive cuspal motion which has a higher rate of recurrent aortic insufficiency [137]. Freedom from reoperation and recurrent AI rates after valve sparing procedures are similar in individuals with both bicuspid and tricuspid aortic valves [136, 137] and these procedures remain an excellent way of treating a pliable BAV and avoiding late complications associated with the implantation of an aortic valve prosthesis.

 The Ross procedure is another good surgical option in a selected group of patients with BAV. Although the Ross procedure has added complexity, the associated morbidity and mortality is relatively low, when performed by experienced surgeons [139, 140]. Freedom from reoperation has been reported to be as high as 99% at 13 years [139]; however, durability of the autograft towards the second postoperative decade is questionable [141]. There are also concerns about progressive dilatation of the pulmonary autograft and subsequent need for reintervention in patients with BAV [142] specifically in male patients with aortic insufficiency and a dilated aortic annulus at the time of surgery [140]. 

## 10. Summary

Over the past decade, research and clinical investigation has better defined our understanding of the pathophysiology of bicuspid aortic valve disease and brought forward an improved appreciation for the heterogeneous phenotypic presentations. Significant gaps in knowledge persist, making optimal management of patients with bicuspid aortic valves and associated aortic aneurysms challenging. Though many patients will inevitably experience significant valvular dysfunction at some point during their lives, the fate of the ascending aorta remains uncertain. The aortopathy associated with BAV disease certainly predisposes individuals to aortic dilatation, aneurysm formation, and aortic dissection; however, it appears that not all BAV aortas behave similarly. Surgical planning should carefully account for negative prognostic risk factors when addressing the bicuspid aortic valve and ascending aorta and tailor operative strategies to maximize long-term results with minimal perioperative morbidity. In the future, specific genetic and molecular markers may help to identify patients at highest risk for aortic complications.

## Figures and Tables

**Table 1:** Prevalence of BAV in previously published investigations.

Author(s)	Study population (*n*)	BAV prevalence (%)	Male : female	Study type	Age (mean ± SD)
Lewis and Grant [1]	215	1.39	3 : 1	Necropsy	—
Wauchope [2]	9966 (6124 males, 3842 females)	0.5	3 : 1	Necropsy	33.6 ± 20.1
Roberts [3]	1,440	0.9	3 : 1	Necropsy	46 (15–79 years)
Larson and Edwards [4]	21,417	1.37	—	Necropsy	—
Datta et al. [5]	8,800	0.59	11 : 1	Necropsy	35.5 (8–70 years)
Pauperio et al. [6]	2,000 (1,499 males, 501 females)	0.65	All males	Necropsy	40.5 ± 20.4 (3 mo–68 years)
Basso et al. [7]	817 (400 males, 417 females)	0.5	3 : 1	2D TTE	Primary school children
Tutar et al. [8]	1,075 (567 males, 508 females)	0.46	4 : 1	2D TTE	Gestational age 38.2 ± 1.9 weeks
Nistri et al. [9]	20,946 (all males)	0.8	N/A	2D TTE	18 ± 2 years

**Table 2:** Fazel-Stanford clusters.

Cluster	Extent of aortic dilatation	Extent of aortic replacement
I	Aortic root alone (13%)	Aortic root
II	Tubular ascending aorta alone (14%)	Supracommissural ascending aorta
III	Tubular ascending aorta and arch (28%)	Supracommissural ascending aorta and transverse arch
IV	Aortic root, tubular ascending aorta, and transverse arch (45%)	Aortic root, ascending aorta, and transverse arch

**Table 3:** Criteria for elective replacement of the ascending aorta in patients with BAV.

AHA/ACC guidelines	
Class I	
(1) Aortic diameter >5.0 cm	
(Level of evidence: B)	
(2) Aneurysm growth rate >0.5 cm/year	
(Level of evidence: B)	
(3) Aortic diameter >4.5 cm with concomitant indication for elective aortic valve repair/replacement	
(Level of evidence: B)	

Aortic size ratios and indexes	
Aortic diameters >4.5 cm and either of the following:	
(1) Ratio of aortic area to body height >10 cm^2^/m in asymptomatic patients with well-functioning BAV, or 8-9 cm^2^/m in symptomatic patients [91]	
(2) Ratio of aortic diameter to body surface area >4.5 cm/m^2^ [92]	

Other criteria (unvalidated)	
Aortic diameters >4.5 cm and any of the following:	
(1) Aortic coarctation, corrected or uncorrected [93]	
(2) First-degree relative with ascending aortic dissection or rupture	
(3) Long smoking history, especially with COPD [94, 95]	
